# Sustainable Conversion of Pulp Industry Sludge into Activated Biochar for High-Performance Methylene Blue Removal

**DOI:** 10.1021/acsomega.5c09623

**Published:** 2026-01-30

**Authors:** Antonio Machado Netto, Marcela de Oliveira Brahim Cortez, José Pedro Rodrigues Ferreira, Renê Chagas da Silva, Leonarde N. Rodrigues, Luciano de Moura Guimarães, Renata Pereira Lopes Moreira

**Affiliations:** † Department of Chemistry, 28120 Universidade Federal de Viçosa (UFV), Av. Peter Henry Rolfs, s/n, Campus Universitário, Viçosa, Minas Gerais 36570-900, Brasil; ‡ Department of Physics, Universidade Federal de Viçosa (UFV), Av. Peter Henry Rolfs, s/n, Campus Universitário, Viçosa, Minas Gerais 36570-900, Brasil

## Abstract

The pulp and paper industry generates large amounts of biological sludge, which can be valorized into activated biochar (A-BC), offering environmental and economic benefits. In this work, A-BCs were produced from this residue using H_3_PO_4_ as an activating agent and subjected to different pyrolysis temperatures (400 to 550 °C). H_3_PO_4_ was selected for its superior activation performance over KOH, enhancing porosity and surface functionalization, while the temperature was chosen to match the main thermal degradation of the sludge’s lignocellulosic matrix. The A-BCs were characterized by proximate and elemental analysis (fixed carbon content ∼30%), FTIR (C=O, O–H, O–Si–O, and P=O functional groups), XRD (predominantly amorphous structure), and Raman spectroscopy (D and G bands). Furthermore, BET surface areas from 7.68 to 1.52 m^2^ g^–1^, a higher heating value (HHV) from 3788 to 4750 kcal kg^–1^, and a point of zero charge (pH_PZC_) from 3.31 to 6.15 were obtained. Increasing the temperature from 400 to 450 °C increases surface area via pore formation, while higher temperatures reduce porosity due to pore collapse and lignin condensation. The A-BC produced at 450 °C (A-BC2) exhibited more than double the surface area and higher methylene blue (MB) removal efficiency than the other samples, consistent with the characterization results. The adsorption assays indicated that the maximum adsorption capacity was 390.73 mg g^–1^, with the Langmuir model fitting the experimental data best (*R* ^2^ = 0.996, *R* ^2^_adj_ = 0.995, and χ^2^ = 1.62). The adsorption kinetics followed the pseudo-second-order model (*R* ^2^ = 0.982, *R* ^2^_adj_ = 0.981, and χ^2^ = 7.78), indicating a chemisorption-controlled mechanism involving electron sharing or exchange between cationic dyes and oxygenated biochar surface groups. The study demonstrates that A-BC from cellulose industry sludge is a viable, sustainable option for dye-containing effluent treatment, supporting circular economy principles.

## Graphical abstract

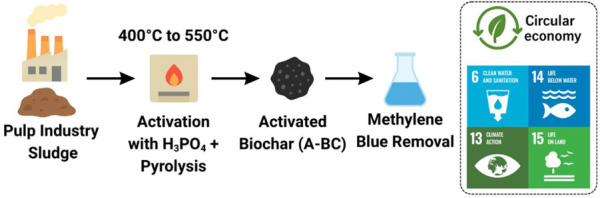

## 1 Introduction

Rapid population growth and technological advancements have led to a significant increase in the generation of solid waste from industrial operations. The pulp and paper industry ranks among the largest worldwide, playing a pivotal role in economic development and enhancing quality of life.^1^ One of the most produced wastes within this industry is the biological sludge (BS) generated during effluent treatment, amounting to 400 million tons annually.^2^ This significant production is due to the enormous consumption of water used in the cellulose extraction process, generating approximately 700 million m^3^ of effluent globally per year.^3^

Landfilling and incineration are common methods for the disposal of this sludge, but landfills face limitations due to high costs, environmental impacts, and public opposition, making them increasingly less viable.^1^ Regarding incineration, this biomass has characteristics that negatively affect the process, such as high moisture content and the presence of inorganics.^4^ Thus, it is necessary to carry out a pretreatment before the combustion of the material for energy production.^5^ In this context, pyrolysis is a promising endothermic thermochemical technology, decomposing organic matter at temperatures from 300 to 600 °C in an oxygen-free atmosphere, producing three fractions: biochar, bio-oil, and biogas.^6^

Additionally, the discharge of residential, industrial, and agricultural effluents has also grown over time; these contain a wide range of contaminants, such as dyes, causing various environmental problems.^7^ Approximately 50,000 tons of untreated organic dyes are discarded annually by industrial sectors.^8^ These contain toxic components, are resistant to degradation, and have carcinogenic potential, posing a threat to aquatic ecosystems and human health.^9–11^

Among dyes, methylene blue (MB) stands out. It is a synthetic dye widely used in the textile, food, cosmetic, and pharmaceutical industries, in addition to having medicinal applications, such as in the treatment of malaria and post-transplant complications.^12,13^ However, its release into water bodies, whether treated or not, can cause serious human health problems, such as cyanosis, tissue necrosis, vomiting, jaundice, and increased heart rate, and it is frequently discarded by industrial processes.^14,15^

Therefore, the removal of MB from wastewater is extremely important for the maintenance of the ecosystem and public health. This challenge has motivated the development of sustainable materials from diverse waste streams. In addition to industrial sludge, agricultural residues have been successfully transformed into advanced materials, such as nanocomposite membranes, for dye removal, providing an environmentally friendly approach to waste valorization.^16^ Several treatment techniques are proposed for this removal, with adsorption being an interesting option, as it is recognized as an economical and sustainable method, valued for its simplified application and low environmental impact.^17^ For this process to occur satisfactorily, the use of a high-efficiency adsorbent material is necessary. In this context, waste-derived materials, including biochar and advanced nanocomposites, have emerged as promising adsorbents, efficiently removing a wide range of organic and inorganic pollutants from wastewater,^7,18,34,39,56^

The production of biochar considers several variables, such as pyrolysis temperature and the activation process, for the production of activated biochar (A-BC). This process improves the structural properties of the material, such as porosity, surface area, and the presence of functional groups, which result in a more efficient adsorbent.^19^ Furthermore, the process temperature also affects these and other characteristics, and the variability of this parameter tends to result in A-BCs with different carbon contents, adsorptive capacities, and hydrophobicities.^20,21^

Therefore, this work aims to advance the valorization of BS from the pulp industry, an underexplored and highly complex industrial residue. Although activated biochar production is an established technology, systematic studies using pulp industry BS, particularly with chemical activation, remain scarce. Such activation can induce physicochemical modifications directly linked to adsorption capacity. Furthermore, the complex composition of this residue makes its effective conversion challenging, yet its successful valorization offers a notable circular economy solution. This work addresses this gap by converting BS into H_3_PO_4_-activated biochar (A-BC) and evaluating the effect of pyrolysis temperature (400–550 °C), a key thermochemical parameter, on the physicochemical properties and adsorption performance of the materials. The biochar with the most favorable characteristics was applied for methylene blue (MB) removal, highlighting the potential of this approach for wastewater treatment.

## 2 Results and Discussion

### 2.1 Synthesis and Characterization of the Biochars

Table 1 presents the results for the composition of the pyrolysis products, the production yield of A-BCs, proximate and elemental analysis, HHV, *S*_BET_, and pore diameter. Regarding the final composition of the pyrolysis products, a higher production of biogas and bio-oil can be observed as the process temperature increases. This behavior is expected due to the greater release of noncondensable and condensable gases, which consequently results in a reduction of the biochar mass produced. The final biochar yields remained close, ranging between 45.27 and 51.79%. These results were higher than those found by Netto et al.,^22^ who reported results ranging from 27.53% to 56.44% for biochars from biological sludge from the pulp industry activated with H_3_PO_4_.

**Table 1:** Results of Pyrolysis Product Composition, A-BC Production Yield, Proximate and Elemental Analyses, HHV, SBET, and Average Pore Diameter^a^

Analysis	Characteristics	Sludge^c^	A-BC1^a^	A-BC2^a^	A-BC3^a^	A-BC4^a^
Final pyrolysis composition	Biochar (%)	-	67.69	63.20	54.84	55.60
	Bio-oil (%)	-	1.35	1.93	5.08	3.04
	Biogas (%)	-	30.96	34.87	40.08	41.36
Final yield^b^	Biochar (%)	-	51.79	45.27	47.31	49.82
Proximate analysis	Moisture (%)	-	6.02	7.99	4.29	8.12
	Volatile matter (%)	71.40	38.85	34.88	25.32	19.93
	Ash (%)	12.40	26.00	26.53	37.75	38.07
	Fixed carbon (%)	16.20	29.13	30.60	32.65	33.88
Higher heating value	HHV (kcal kg^–1^)	4566	4617	4750	3852	3788
*S*_BET_	m^2^ g^–1^	-	3.54	7.68	3.61	1.52
Average pore diameter	Nm	-	6.25	1.92	1.92	1.91
Pore volume	cm^3^ g^–1^	-	0.0207	0.0228	0.0142	0.0099
Elemental analysis	C (%)	43.01	40.35	44.82	49.83	39.29
	H (%)	6.43	2.86	4.20	4.33	2.19
	N (%)	4.65	3.73	5.14	5.22	3.74
	S (%)	3.09	0.70	1.45	1.26	0.55
	O (%)	42.82	52.36	44.39	39.37	54.23
	H/C	1.79	0.85	1.12	1.04	0.67
	O/C	0.75	0.97	0.74	0.59	1.04

a Production temperature of A-BC. A-BC1 (*T* = 400 °C); (b) A-BC2 (*T* = 450 °C); (c) A-BC3 (*T* = 500 °C); and (d) A-BC4 (*T* = 550 °C).

b Total process yield, in relation to the mass production of A-BC, after washing.

c Proximate analysis performed on a dry basis—analysis not performed. -Not applicable.

Regarding the proximate analysis, a reduction in the volatile matter content of the biochars is observed compared to the raw sludge due to the pyrolysis process, in addition to a progressive decrease in this parameter as the pyrolysis temperature increases. This behavior occurs due to the greater release of volatile substances during the process at higher temperatures.^23^ The ash content and fixed carbon show the opposite behavior, with an increase in their content as the temperature increases. These results demonstrate the retention of inorganics while the organic fraction is decomposed and released through volatilization, and a trend toward greater efficiency for compound removal in adsorption processes, respectively.^22,24^

The higher heating value of A-BC1 and A-BC2 was higher than that of the sludge, indicating that the carbonization process at lower temperatures increased the energy potential of these materials. These biochars showed an the higher heating value (HHV) higher than that of wood, as found by Sseremba et al.,^25^ who obtained a result of 4047 kcal kg^–1^ for *Eucalyptus grandis*. On the other hand, A-BC3 and A-BC4, which were pyrolyzed at higher temperatures, showed lower HHVs than the biomass, due to greater losses of carbon structures in the material.^26^ However, despite this reduction, these A-BCs showed higher HHVs than those found by Ribeiro et al.,^27^ who obtained results ranging from 1410.1 to 1816.4 kcal kg^–1^ for biochars from cosmetics industry sludge.

The A-BCs were subjected to N_2_ adsorption/desorption analysis, and the isotherms can be observed in Figure 1. The isotherms are of type IV­(a), as they exhibited capillary condensation accompanied by hysteresis.^28^ The BET specific surface area (*S*_BET_) results found for the A-BCs were between 1.52 and 7.68 m^2^ g^–1^. When the temperature increases from 400 to 450 °C, an increase in the surface area is observed, likely due to the formation of pores during the release of volatile matter; however, this area decreases at higher temperatures as a consequence of pore collapse or fusion.^29^ According to Ma et al., this behavior is consistent with biomass thermal treatments, in which excessive thermal severity leads to structural collapse and lignin condensation, compromising the porous network.^30^

**Figure 1:**
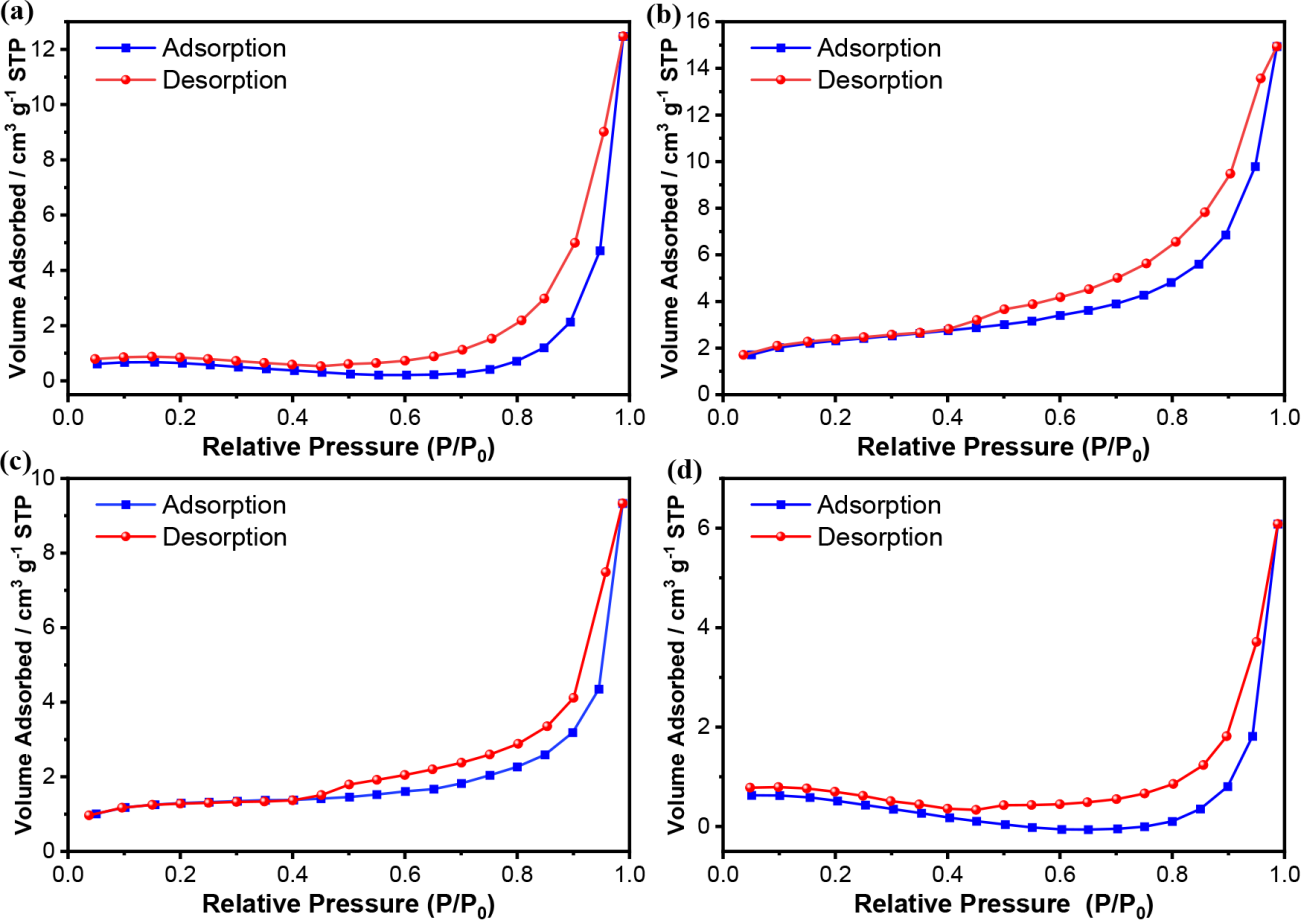
Nitrogen adsorption and desorption isotherms of the A-BCs obtained from cellulose industry sludge. (a) A-BC1 (*T* = 400 °C); (b) A-BC2 (*T* = 450 °C); (c) A-BC3 (*T* = 500 °C); and (d) A-BC4 (*T* = 550 °C).

A similar result regarding *S*_BET_ was found by Netto et al.^22^ in the production of biochar from biological sludge from the cellulose industry activated with H_3_PO_4_, with an area of 3.34 m^2^ g^–1^. In a previous study by our group, approximately 30 biochars were produced from the same biomass using phosphoric acid or potassium hydroxide as activating agents (Netto et al., 2021 ^22^). The phosphoric acid-activated biochars consistently exhibited yields between 33 and 44%, matching the values obtained in the present study and confirming the reproducibility of the material across different synthesis batches.

Furthermore, the average pore diameters were determined, with mesopores (2–50 nm) being observed for A-BC1 and micropores (less than 2 nm) for the others, classified according to IUPAC (International Union of Pure and Applied Chemistry). A similar result was found by Salgado et al.^31^, who obtained biochars with average pore diameters of 1.67 nm. Additionally, the pore volume data directly support the *S*_BET_ results. A-BC2 showed the highest pore volume, indicating that the porous structure was most developed at 450 °C, before decreasing at higher pyrolysis temperatures.

The elemental analysis shows a reduction of hydrogen in the A-BCs compared to the biological sludge, due to the pyrolysis process and the resulting release of significant rates of biogas.^32^ The reduction of the H/C ratio is an indication of the carbonization process. Sulfur showed a similar behavior, which favors the subsequent use of the biochars for energy production through combustion, minimizing the release of greenhouse gases. The Van Krevelen diagram (Figure 2a) indicates that all the activated biochars (A-BCs) retained oxygen in their structure, possibly due to the significant presence of O–Si–O and P=O bonds. This hypothesis is corroborated by the Fourier-transform infrared spectroscopy (FTIR) results (Figure 2b), which showed more intense bands associated with these bonds.^33,34^

**Figure 2:**
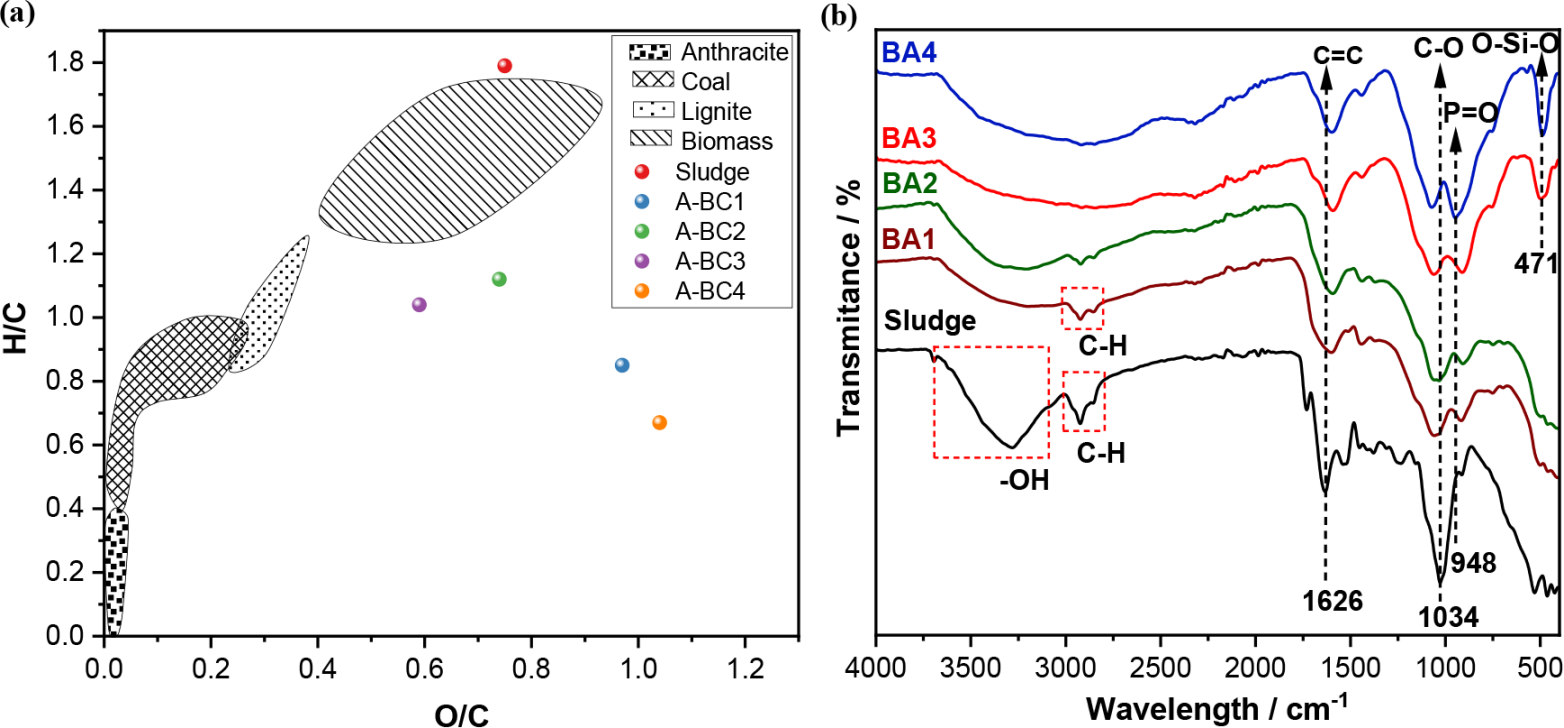
(a) Van Krevelen diagram and (b) FTIR of the industrial sludge (BS) and the produced activated biochars (A-BCs). A-BC1 (*T* = 400 °C); A-BC2 (*T* = 450 °C); A-BC3 (*T* = 500 °C); and A-BC4 (*T* = 550 °C).

Regarding the FTIR analysis, bands are observed in the regions of 1626 and 1034 cm^–1^ for the sludge and for all A-BCs, referring to the stretching of C=C and C–O bonds, respectively.^35,36^ The C=C bond indicates the presence of aromatic compounds, while the shift of the C–O bands in the A-BCs indicates a modification in the material’s structure during the formation of the biochars. Furthermore, a reduction of bands is observed in the region between 2990 and 2825 cm^–1^, related to the presence of C–H bonds, indicating the elimination of lipid components, the main precursors of bio-oil, and denoting the incomplete carbonization of A-BC1.^36^ The bands between 3700 and 3000 cm^–1^, corresponding to O–H stretching, are significantly reduced in the A-BCs compared to the sludge, as these groups—found in water, alcohols, and other compounds—are released during pyrolysis.^32,37^ Moreover, bands are observed in the 471 cm^–1^ region for A-BC3 and A-BC4, referring to the stretching of the O–Si–O bond, indicating a greater interaction of the material with silica at higher pyrolysis temperatures.^34^ Similarly, a shift of bands toward the 948 cm^–1^ region is visible as the temperature increases. This behavior indicates the presence of P=O bonds in the material, especially in A-BC4, suggesting a better connection of phosphate groups with the material.^33^

The X-ray diffraction (XRD) pattern, shown in Figure S1, shows a broad peak in the region between 15° and 30°, characteristic of amorphous materials. However, peaks can be observed at 20.6° and 26.6°, attributed to the presence of quartz (SiO_2_).^22^ The first peak indicates high crystallization of quartz, while the second, present only in A-BC3 and A-BC4 (produced at higher pyrolysis temperatures), reflects a greater interaction of silica with the material, as confirmed by FTIR.^38^ Furthermore, a peak can be observed near the 12.1° region, characteristic of kaolinite (Al_2_O_3_·2SiO_2_·2H_2_O), which is a clay mineral widely used in the bleaching of cellulosic pulp.^39^

The Raman spectra of the A-BCs were processed by baseline subtraction, followed by deconvolution to resolve overlapping bands, and are presented in Figure 3, showing two main bands. The D band, at 1327, 1350, and 1360 cm^– 1^, indicates defects in the carbon structure, while the G band, at 1550, 1560, and 1580 cm^– 1^, is linked to graphitic carbon, representing the stretching of bonds between sp^2^ carbon atoms in rings and chains.^40^ The *I*_D_/*I*_G_ ratio was also considered, as it reflects the degree of structural disorder in carbon materials. Ratios close to 1 indicate higher disorder, a characteristic that favors adsorption performance.^41,42^ This ratio varied from 1.10 to 1.26 for A-BC2, A-BC3, and A-BC4, demonstrating the promising potential of these adsorbents. A progressive increase in the *I*_D_/*I*_G_ ratio was observed with increasing pyrolysis temperature, suggesting the development of defective fused aromatic ring structures.^43^ However, it is possible to observe in Figure 3a, referring to A-BC1, that the D band shows a reduced peak size, generating an *I*_D_/*I*_G_ ratio of 0.83. This behavior may be related to the remaining organic matter in this material, as corroborated by FTIR, which causes fluorescence in the spectrum.

**Figure 3:**
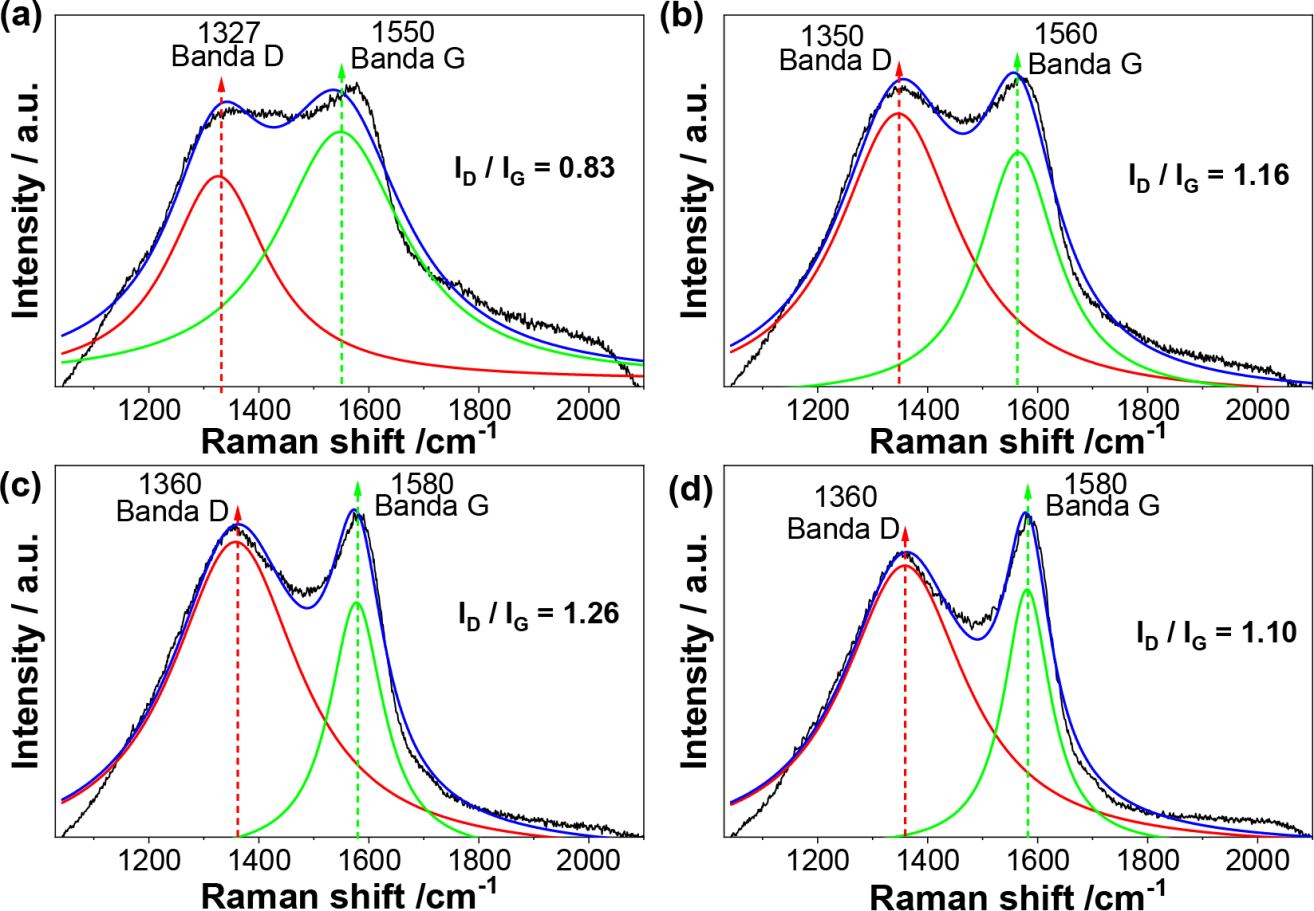
Raman spectra of the biochars produced by the pyrolysis of biological sludge. (a) A-BC1 (*T* = 400 °C); (b) A-BC2 (*T* = 450 °C); (c) A-BC3 (*T* = 500 °C); and (d) A-BC4 (*T* = 550 °C).

The Scanning Electron Microscopy (SEM) images of the sludge and the biochars can be observed in Figure 4. It is possible to observe the change in the material’s morphology. While the sludge presents a smoother and more compact surface, as the pyrolysis temperature increases, the morphology becomes rougher, with a granular appearance, showing multiplanar structures and the presence of cavities. The pyrolysis temperatures, along with the presence of the H_3_PO_4_ activator, promote the elimination of moisture and volatile compounds and the generation of oxides and free radicals on the biochar surface, culminating in an increase in its roughness.^36^

**Figure 4:**
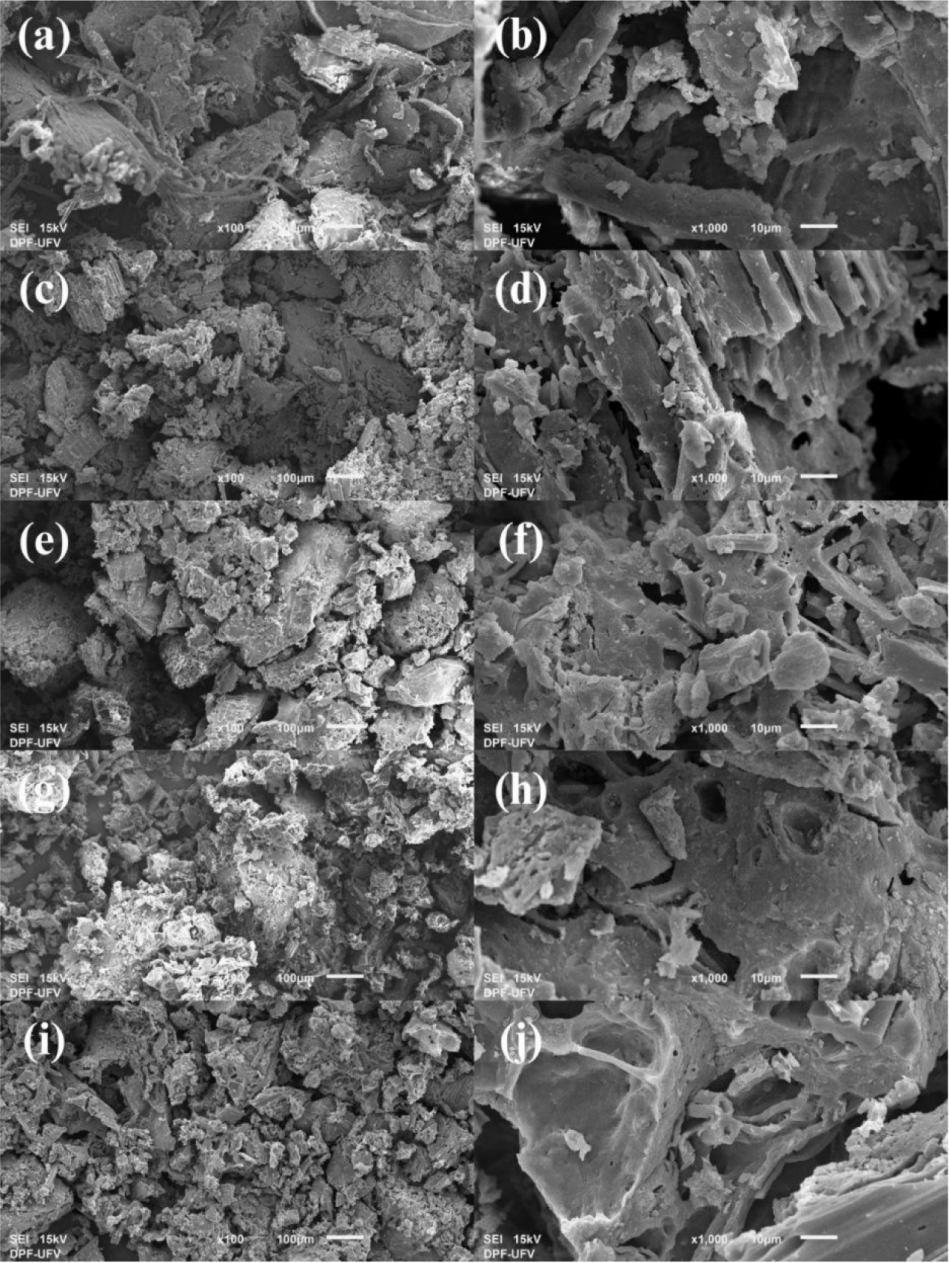
Scanning Electron Microscopy (SEM) images: (a) and (b) sludge; (c) and (d) A-BC1; (e) and (f) A-BC2; (g) and (h) A-BC3; (i) and (j) A-BC4, with 100× and 1000× magnifications, respectively.

From the EDS analysis (Table S1 and Figure S2), including elemental mapping (Figures S3–S7), disregarding carbon and copper, the presence of high oxygen contents is noted, which is characteristic of functional groups, as confirmed by FTIR. The presence of Ca, Na, Al, and Si, originating from the biomass, was also observed. These elements are characteristic of the cellulose production process, the effluent treatment, and impurities from the industry’s raw material, respectively. The presence of P only in the biochars is consistent with the material’s activation process.

The results of the thermogravimetric analysis (TGA) are shown in Figure S8, which displays the TGA and DTG curves for the activated biochars and the biological sludge. A common thermal event can be seen in the materials, the first being between 25 and 150 °C, attributed to moisture loss.^32^ Other events occur between 250 and 300 °C, 300 and 400 °C, and 400 and 600 °C, related to the decomposition of hemicelluloses, cellulose, and lignin, respectively.^44^ It can be observed that greater losses, with more intense DTG peaks, are observed for the sludge and A-BC1 (Figure S8e,a), which present noncarbonized organic matter, as proven by FTIR. Similar results were observed by Monteiro et al.^36^ for biochars derived from cosmetics industry sludge activated with phosphoric acid and pyrolyzed at 450 and 550 °C.

Figure S9 presents the point of zero charge (pH_PZC_) values for the biochars. The results indicate a decreasing trend of the pH_PZC_ with the increase in pyrolysis temperature, except for A-BC2, which exhibited a higher value than the others. From the pH_PZC_, it is possible to predict the adsorption behavior of the A-BCs, such that at pH > pH pH_PZC_ the material tends to acquire a negative charge, while at pH < pH pH_PZC_ it tends to acquire a positive charge.^45^ Guo et al.^46^ found similar pH pH_PZC_ results for sludge from a paper mill, ranging from 4.0 to 6.0, which demonstrates the tendency for acidic properties in this type of material.

To evaluate the presence of elements such as trace heavy metals originating from the pulp industry sludge, the elemental composition of the sludge and the produced A-BCs was determined by ICP-OES (Table S2). The analysis confirms that critical toxic heavy metals, including cadmium (Cd), chromium (Cr), mercury (Hg), nickel (Ni), and lead (Pb), were all below the limit of quantification (<LoQ) of the analytical method in both the original sludge and all biochars, indicating their minimal presence in these materials. This finding is consistent with the analysis of the raw effluent from which this sludge is derived, as reported by Netto et al.,^22^ whose data also showed these heavy metals were < LoQ. However, high concentrations of sodium (Na) were detected in both the biochars and the raw sludge itself. This indicates that the high sodium content is an intrinsic characteristic of the feedstock, likely attributed to the extensive use of sodium-based chemicals during the cellulose pulping process, which subsequently accumulate in the biological sludge.

### 2.2 Adsorption of Methylene Blue

Initially, the adsorptive capacity of the activated biochars (A-BCs) was screened, with results shown in Figure S10. A-BC2 (produced at 450 °C) exhibited superior performance compared to the others, correlating directly with its physicochemical properties. It displayed a markedly higher specific surface area, suggesting that at 450 °C, volatile matter release promotes pore formation, whereas at 500–550 °C, pore collapse or fusion reduces available surface area. A-BC2 also presented a distinct surface chemistry, with a pH_PZC_ of 6.15, higher than A-BC1 and deviating from the acidification trend at higher temperatures. Its adsorption capacity was comparable to commercial activated carbon, highlighting its potential for MB removal. Consequently, A-BC2 was selected for subsequent assays.

Thus, the effect of the variation in the A-BC2 dosage on the removal of MB was investigated, and the results are shown in Figure 5a. It is observed that the dosage of 1.50 g L^–1^ showed the best performance, with statistical significance confirmed by ANOVA (*p* < 0.05), resulting in an adsorption capacity of 318.01 ± 5.66 mg g^–1^ and a removal of 95.5% of the adsorbate. Zhao et al.^47^ found similar results in the removal percentage of MB (95.1%) with a dosage of 1.5 g L^–1^ of poplar leaf biochar; however, the adsorption capacity results were much lower, at approximately 8.12 mg g^–1^.

**Figure 5:**
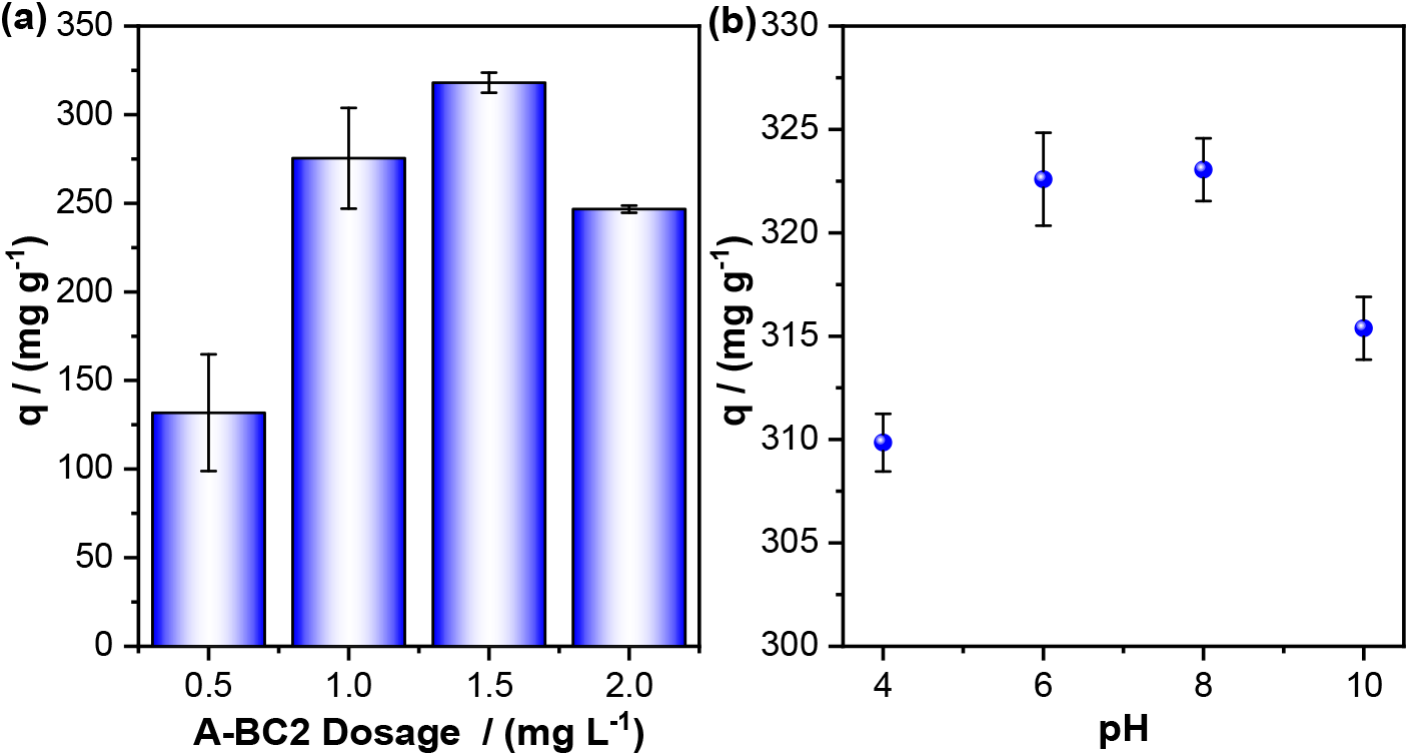
Removal of methylene blue using A-BC2. (a) Effect of A-BC2 dosage; (b) effect of initial pH. Experimental conditions: room temperature (∼25 °C), adsorption time 1444 min, agitation: ∼180 rpm, [MB] = 100 mg L^–1^.

Figure 5b shows the effect of initial pH on the adsorption of MB by A-BC2. The highest adsorption capacities were obtained at pH 6 and 8, with values of 322.59 ± 2.25 and 323.05 ± 1.52 mg g^–1^, respectively. Based on the statistical analysis (ANOVA, *p* < 0.05) and the low standard deviations, it was determined that pH 8 is the most suitable for the adsorption process. This finding aligns with the pH_PZC_ of A-BC2 (6.15) and the p*K*_a_ of MB (3.8), as expected. Thus, at pH 8, the adsorbent surface is deprotonated (negatively charged), while the dye remains in its cationic form, favoring the electrostatic interaction between them.^48^ However, a reduction in adsorption capacity is noted at pH 10. This behavior may occur due to functional groups possibly present on the A-BCs, such as −COOH and −OH, which deprotonate at highly alkaline pHs (pH > 8) to become −COO^–^ and −O^–^, causing electrostatic repulsion between the groups and consequently a reduction in adsorption.^49^ This pH-dependent surface charge plays a fundamental role in adsorption. Similarly, Adaileh et al.^50^ reported that the adsorption of anionic bicarbonate reached its maximum at pH 7.5, corresponding to the point of highest positive surface charge on their nanocomposite material.

Figure 6a presents the kinetic study results, indicating that adsorption equilibrium was achieved after 1200 min. This outcome corroborates the study of Lee, Fiore e Berruti (2024), who observed equilibrium within 1000–1200 min according to the model that best fitted their data. However, in our study, the adsorption capacity was significantly higher, reaching approximately 366 mg g^–1^, whereas those authors reported values close to 90 mg g^–1^. This longer adsorption time may be related to the average pore size, since the material falls into the micropore category; due to their small size, these pores impede a faster diffusion rate of dye molecules to the adsorption sites.^51^ Despite this, this microporous structure is fundamental to the high capacity, providing sites that are dimensionally appropriate for the efficient capture of MB molecules. This is consistent with other high-performance composites, such as the ACTF@ZIF-8 reported by Hemdan et al.,^52^ which also relies on a bimodal structure including micropores (1.5–2.0 nm) to achieve high MB removal efficiency.

**Figure 6:**
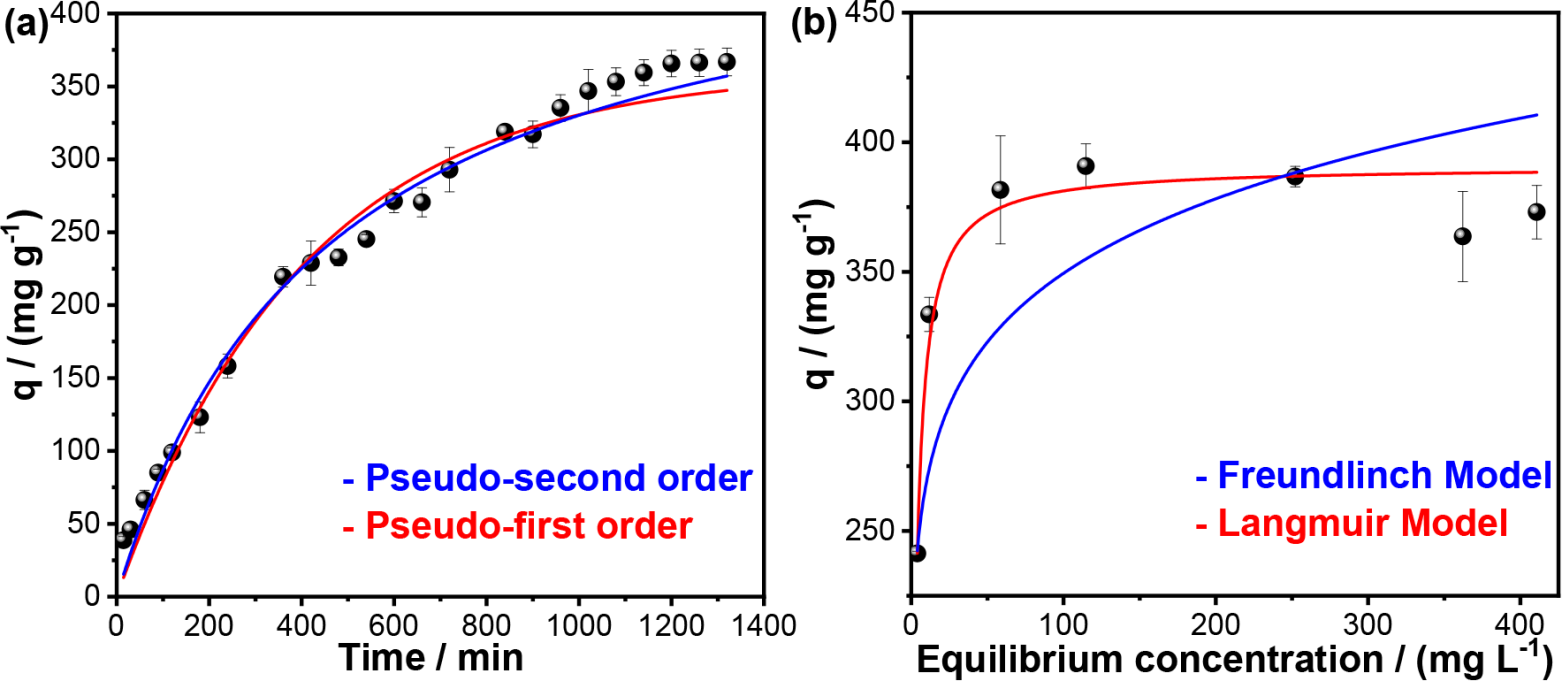
(a) Kinetic study and (b) adsorption isotherm of methylene blue by A-BC2. Experimental conditions: temperature: ∼25 °C, pH = 8, agitation: ∼180 rpm, and adsorbent dose: 1.50 g L^–1^.

The experimental data were fitted to the pseudo-first-order and pseudo-second-order kinetic models, the parameters of which are shown in Table 2. It is noted, through the *R* ^2^, *R* ^2^_adj_ and χ^2^, values, that the pseudo-second-order model better fit the data, indicating that the adsorption process is predominantly controlled by chemisorption.^53^ This mechanism involves electron sharing or exchange between cationic dye molecules and oxygenated groups on the biochar surface, rather than weak van der Waals interactions.^54^

**Table 2:** Kinetic Model Parameters for Methylene Blue Adsorption^a^

Model	*q*_e_ (mg g^–1^)	*k*_1_ (min^–1^)	*k*_2_ (g mg^–1^min^–1^)	*R* ^2^	*R* ^2^_adj_	χ2	*p*-value
Pseudoprimeira ordem	361.20	0.0025	-	0.973	0.972	12.01	8.82 × 10^–22^
Pseudosegunda ordem	479.43	-	4.615	0.982	0.981	7.78	1.58 × 10^–23^

a *p*-value = probability of a given statistical measure.

The adsorption isotherm results and fitted model parameters are presented in Figure 6b and Table 3. The Langmuir model provided a superior fit, with higher *R* ^2^, *R* ^2^ adj, and χ^2^ values, indicating predominantly monolayer adsorption and a relatively homogeneous distribution of active sites.^31^ Under these conditions, a *q*_max_ of 390.73 mg g^–1^ was obtained—a notably high value considering the modest BET surface area of 7.68 m^2^ g^– 1^. This can be attributed to the adsorption of aromatic dyes, such as methylene blue, which involves not only chemisorption via surface functional groups but also π–π and π donor–acceptor interactions.^53^ These interactions enhance local affinity between MB molecules and the functionalized or aromatically conjugated active sites, enabling high adsorption capacity even in materials with moderate or low surface area.

**Table 3:** Isotherm Parameters for Methylene Blue Adsorption^a^

Model	*q*_max_ (mg g^–1^)	*k*_L_ (L g^–1^)	k_F_(mg^1‑(1/n)^ g^–2^ L^1/n^)	*N*	*R* ^2^	*R* ^2^_adj_	χ^2^	*p*-value
Langmuir	390.73	0.3992	-	-	0.996	0.995	1.62	7.68 × 10^–11^
Freundlich	-	-	206.684	8.771	0.935	0.922	25.11	7.30 × 10^–8^

a *p*-value = probability of a given statistical measure.

The *q*_max_ value indicates that A-BC2 is competitive with adsorbents derived from similar wastes and agricultural biomasses reported in the literature. For instance, Qin et al.^54^ and Fan et al.^55^ reported lower *q*_max_ values for methylene blue removal using HNO_3_-activated biological sludge biochar (176.05 mg g^–1^) and *Artocarpus heterophyllus* biochar (127.44 mg g^– 1^), respectively. Lee et al.^48^ achieved a higher *q*_max_ of 558.95 mg g^–1^ using ZnCl_2_-modified biochar from pulp and paper sludge; however, their material had a much larger surface area (877.25 m^2^ g^–1^), likely due to the ZnCl_2_ activation. These results suggest potential for exploring this activator in future studies using the same residue.

To assess the reusability and stability of A-BC2, three consecutive adsorption/desorption cycles were performed. The material maintained high performance, with MB removal efficiencies of 72.17 ± 5.78%, 99.04 ± 0.31%, and 93.69 ± 1.81% for the first, second, and third cycles, respectively. Similar behavior was reported by Zhou et al.,^56^ who observed an unexpected increase in efficiency between the first and second cycles using a NaOH-regenerated, iron-decorated biochar for methyl orange removal, likely due to activation of the material during regeneration. The slight decrease between the second and third cycles can be attributed to progressive saturation of surface functional groups and partial blockage of active sites after repeated use.^57^

This elevated adsorption is comparable to other advanced composite materials. Lawtae and Tangsathitkulchai^51^ reported that a chitosan–*Ocimum basilicum* leaves–ZnO (ChOBLZnO) composite membrane maintained 83.92% removal efficiency after five cycles. Similarly, Lee et al.^48^ achieved over 90% MB removal with ZnCl_2_-activated biochar from pulp and paper sludge in consecutive cycles. A-BC2 also maintained significant removal efficiency after three cycles, confirming its stability and potential for repeated use. These results demonstrate that A-BC2 offers competitive performance among sustainable adsorbents, supporting its applicability for dye removal.

The adsorption isotherms at different temperatures for the thermodynamic study are shown in Figures 6b and S11. The calculated thermodynamic parameters were Δ*G*° = 2.28 kJ mol^–1^, Δ*H* = 34.67 kJ mol^–1^, and Δ*S* = 0.11 kJ K^–1^ mol^–1^. Although the slightly positive Δ*G*° suggests that the adsorption is nonspontaneous under standard conditions, the positive Δ*S* and endothermic nature (Δ*H* > 0) indicate that the process becomes favorable at the experimental temperatures, with increased randomness at the solid/solution interface.^58^ The activation energy (Ea) was determined to be 79.85 kJ mol^–1^, consistent with chemisorption, since physisorption typically exhibits Ea between 5 and 40 kJ mol^–1^, while chemisorption ranges from 40 to 800 kJ mol^–1^.^59^ These results confirm that chemisorption is the predominant adsorption mechanism.

Boehm titration was performed on A-BC2, used for MB removal, revealing 1.25 mol g^–1^ of carboxylic groups, 0.81 mol g^–1^ of lactone/lactol groups, and 1.44 mol g^–1^ of phenolic groups. These results confirm the presence of surface functionalities directly involved in the adsorption process, which is predominantly driven by chemisorption. This supports that the high adsorption capacity is primarily due to interactions mediated by functional groups on the biochar surface.^53^

### 2.3 Estimated Production Costs

The estimated production costs of the activated biochars (A-BC1–A-BC4) are presented in Table S3. These values provide a simplified estimation based on process variables and do not represent a full techno-economic analysis, as factors such as labor, equipment efficiency, depreciation, and large-scale integration were not included. Total costs ranged from US$ 0.60 to 0.74 kg^– 1^, with H_3_PO_4_ (85%) as the main contributor (>60%), followed by energy for drying and pyrolysis. A-BC2 had the highest cost (US$ 0.74 kg^–1^) due to its lower pyrolysis yield but offers superior adsorption performance, balancing cost and efficiency.

This trend of increased cost for chemically activated biochars is consistent with literature reports for other feedstocks, such as Si-modified oiltea camellia shell biochars (0.67–1.44 USD·kg^–1^)^60^ and FeCl_3_-activated sugar cane bagasse biochars (0.02–0.06 USD·g^–1^),^61^ with broader ranges of 0.56–5.49 USD·kg^–1^ depending on precursor and activation [62–64]. The costs reported here fall at the lower end of these ranges, highlighting the relative economic viability of phosphoric-acid-activated biochars from industrial sludge. These results suggest that, when integrated into existing industrial processes, such biochars can provide a sustainable and low-cost adsorbent for wastewater treatment.

## 3 Conclusion

Therefore, in this work, activated biochars were produced from biological sludge of the cellulose industry using phosphoric acid as an activating agent, with pyrolysis temperatures ranging from 400 and 550 °C. The physicochemical characterization of the materials showed that the increase in temperature promoted significant changes in the biochar’s structure, including greater biomass carbonization and an increase in porosity and specific surface area, which are determining factors for improving the material’s adsorption capacity. The results indicated that the biochar produced at 450 °C (A-BC2) showed the best adsorption capacity for the removal of methylene blue, reaching a *q*_*max*_ of 390.73 mg g^–1^, according to the Langmuir model. The adsorption assays demonstrated that the material’s efficiency is directly related to the pyrolysis temperature, the adsorbent dosage, and the solution pH, with pH 8 and a dosage of 1.50 g L^–1^ being the most suitable parameters for dye removal. Thus, the use of cellulose industry sludge for the production of activated biochar proved to be a viable and sustainable alternative for the treatment of dye-containing effluents. Furthermore, the valorization of this industrial waste is aligned with the principles of the circular economy, promoting the reuse of materials and the reduction of environmental impacts. Previous work by our group showed that a biochar produced under conditions similar to A-BC2 remained highly stable in complex industrial effluent, demonstrating the broad applicability of the material in real wastewater treatment.

## 4 Experimental Section

### 4.1 Reagents and Solutions

The reagents used in this work were of analytical grade. Phosphoric acid 85% (CAS 7664-38-2), sodium hydroxide 98.93% (CAS 1310-73-2), and methylene blue (CAS 122965-43-9) were obtained from NEON, and commercial powdered activated carbon (CAS 7440-44-0) was obtained from Nuclear Comércio e Representações Ltd., São Paulo, Brazil. All solutions were prepared using ultrapure water, obtained using a Milli-Q system (Millipore, Bedford, MA, USA).

### 4.2 Biological Sludge (BS)

The biomass (BS) was obtained from a pulp and paper industry in Minas Gerais, Brazil. The BS was collected after biological treatment, having a consistency of 12.84%, which means that 12.84% of the sludge mass is composed of solids (organic and inorganic), and the remainder (87.16%) is water. The material was kept under refrigeration to prevent the decomposition of organic matter and the proliferation of microorganisms.

### 4.3 Synthesis of the Biochars

For the synthesis of the A-BCs, the biomass was impregnated with H_3_PO_4_ at a mass ratio (mass of activating agent/mass of biomass) of 0.40. H_3_PO_4_ and the selected mass ratio were chosen based on an optimization study by Netto et al.^22^ using the same pulp industry sludge, which demonstrated that phosphoric acid activation outperformed KOH activation for this material. Additionally, H_3_PO_4_ was selected for its ability to promote the development of a porous structure and the formation of phosphorus and oxygen containing functional groups, enhancing the surface functionalization and adsorptive capacity of the activated carbon.^55^ Subsequently, the mixture was dried in an oven at 105 °C for 15 h.

The material was pyrolyzed in a custom reactor (Figure S12) under N_2_ flow (13.5 L h^–1^) at 20 °C min^–1^ for 54 min, with final temperatures of 400–550 °C. Although the heating rate can influence pore structure, it was kept constant to focus on the effect of final pyrolysis temperature, and a note summarizing this has been added to the manuscript. This temperature range was selected to cover the main thermal decomposition of the sludge’s lignocellulosic matrix, particularly the degradation of cellulose and lignin, as reported by Zhou et al.^56^ The bio-oil (BO) was collected, and its yield was analyzed, while the biogas (BG) was quantified by mass balance. After the reactor cooled, the A-BCs were recovered and sieved to 32 mesh.

Finally, the A-BCs were washed in two steps according to a methodology adapted from Reddy et al.^57^ First, the material was placed in contact with a NaOH solution (0.5 mol L^–1^) for 30 min, and then with distilled water for 15 min. This process was repeated until the pH was close to neutral, also aiming to remove residual ash. Subsequently, the biochars were dried in an oven for 15 h at 105 °C. The pyrolysis yield was calculated according to eq 1

1 $$R\left(\%\right)=\left(\frac{m_{P}}{m_{T}}\right)\times 100$$

Where: *R* is the yield (%); *m*_*P*_ is the mass of the produced material (g); *m*_*T*_ is the initial mass before pyrolysis (g).

### 4.4 Characterization of the Materials

The A-BCs were initially characterized by the parameters of moisture, ash, volatile matter, and fixed carbon.^58^ The higher heating value was determined using a bomb calorimeter (IKA, C200, Germany), following the calorimetric methodology.^59^ Elemental analysis^60^ was performed using a LECO—TruSPec Micro instrument to determine the contents of C, H, N, and S, with the oxygen content being obtained by subtracting the sum of the other elements from 100%. The BET specific surface area (*S*_BET_) was determined from nitrogen adsorption and desorption isotherms obtained using a BET analyzer (NOVA 600 Anton Paar). Before the measurements, the samples were subjected to a degassing step at 393 K for 4 h. Subsequently, they were treated with N_2_ gas at 353 K for 4 h. Finally, the *S*_BET_ was calculated by the Brunauer–Emmett–Teller (BET) method.

Fourier-transform infrared spectroscopy analysis was performed using an ALPHAA II instrument (BRUKER, USA) equipped with an attenuated total reflectance (ATR) accessory. The analysis consisted of 16 transmittance scans in the 400 to 4000 cm^–1^ range at a spectral resolution of 4 cm^–1^. X-ray diffraction analysis was performed on a Bruker (D8 Discover) diffractometer equipped with a copper tube and a Goebel mirror. Raman spectroscopy was performed on a Renishaw InVia instrument, equipped with a 50x Olympus Bx41 objective lens and an Nd:YAG laser at λ = 514 nm. Scanning Electron Microscopy analysis was performed using a FIB–Quanta FEG 3D FEI microscope (FEI Company, Hillsboro, OR, USA), which was coupled with Energy Dispersive Spectroscopy (EDS) for inorganic analysis.

Thermogravimetric analysis was performed on a Shimadzu instrument (DTG-60H, Japan) by heating the samples from 24 to 800 °C at a rate of 10 °C min^–1^ under an inert N_2_ atmosphere with a flow of 50 mL min^–1^. The point of zero charge was determined according to the methodology adapted from De Souza et al.^61^ Metal analysis was conducted using inductively coupled plasma optical emission spectrometry (ICP-OES) on an Agilent 5110 instrument (USA). Samples were digested with 50 mg of material, 10 mL H_2_SO_4_, and 5 mL H_2_O_2_, then diluted 10-fold for measurement. Quantification was performed in semiquantitative mode using a 5 mg L^–1^ multielement internal standard to correct for drift and normalize the instrumental response.

Boehm titration was performed on the A-BC exhibiting the highest adsorption capacity for methylene blue to qualitatively and quantitatively identify surface functional groups.^62^ Following the method adapted from Barbosa et al.,^63^ 0.1 g of biochar was added to 25 mL of 0.1 mol L^–1^ NaHCO_3_, Na_2_CO_3_, or NaOH solutions and agitated for 24 h at 180 rpm and 25 °C. After filtration, 10 mL of the supernatant was titrated with 0.1 mol L^–1^ HCl using phenolphthalein as an end point indicator. Control experiments without biochar allowed determination of acidic sites by the difference in HCl consumption. Carboxylic groups were quantified via NaHCO_3_, lactone/lactol groups via Na_2_CO_3_, and phenolic groups via NaOH.^64^

### 4.5 Adsorption Assays

The adsorption assays were conducted in Erlenmeyer flasks with different dosages of the biochar in 50 mL of a MB solution (500 mg L^–1^), and the initial pH was adjusted with NaOH or HCl solutions, both at 0.100 mol L^–1^. The system was agitated at 105 rpm at room temperature (∼25 °C) for 1140 min. Subsequently, the system was subjected to centrifugation (10 min at 4000 rpm) to remove the supernatant, and the remaining concentration of MB was quantified by UV–vis spectrophotometry in scanning mode.

Initially, a preliminary assay was performed with all four biochars and the commercial activated carbon (AC), using a dosage of 1.00 g L^–1^ of the A-BCs and AC, the natural solution pH of 5.7, a contact time of 1440 min, and a 100 mg L^–1^ concentration of methylene blue. This was done to determine which of the A-BCs presented the best results in dye removal, in order to use it in subsequent tests, and to compare its performance with that of the AC.

Two assays were performed to optimize the A-BC dose and the pH. Thus, the following were evaluated: (1) biochar dosages of 0.50, 1.00, 1.50, and 2.00 g L^–1^, while maintaining the natural solution pH of 5.7; and (2) the initial pH, evaluating values of 4, 6, 8, and 10, using the dose determined in the first assay. Both tests used a contact time of 1440 min and a concentration of methylene blue of 500 mg L^–1^.The results were evaluated using the adsorption capacity, which was determined according to eq 2

2 $$q=\frac{VC_{in}-VC}{m_{ads}}$$

Where: *q* is the adsorption capacity (mg g^–1^), *V* is the volume of the solution (mL), *C*_*in*_ is the initial concentration of the solution (mg L^–1^), *C* is the concentration of the solution (mg L^–1^) at equilibrium, and *m*_*ads*_ is the mass of the adsorbent (g).

The kinetic assays were performed in batch using the previously defined adsorbent dose and pH, with 50 mL of MB (500 mg L^–1^). Aliquots were collected at time intervals ranging from 15 to 1440 min. The experimental data were fitted to the pseudo-first-order and pseudo-second-order kinetic models, as described in eqs 3 and 4.

3 $$q_{t}=q_{e}\left[1-exp^{-k_{1}t}\right]$$

4 $$q_{t}=\frac{k_{2}{\;q}_{e}^{2}\;t}{1+k_{2}{\;q}_{e}\;t}$$

Where: *q_e_* and *q_t_* are the amounts adsorbed per gram of adsorbent at equilibrium and at time *t* (min), respectively, in mg g^–1^; *k*_1_ is the pseudo-first-order adsorption rate constant (min^–1^); *k*_2_ is the pseudo-second-order adsorption rate constant (g mg^–1^ min^–1^);

The adsorption isotherms were obtained using the previously defined adsorbent dose, pH, and equilibrium time, with MB concentrations ranging from 250 to 1000 mg L^–1^ at 25 °C. The experimental data were fitted to the Langmuir and Freundlich models, described in eqs 5 and 6, respectively.

5 $$q_{e}=\frac{q_{max}K_{L}{\;C}_{e}}{1+K_{L}{\;C}_{e}}$$

6 $$q_{e}=K_{F}C_{e}^{\frac{1}{n}}$$

Where: *q_max_* is the maximum adsorption capacity (mg g^–1^); *K_L_* is the adsorbate/adsorbent interaction constant (L mg^–1^); *C_e_* is the adsorbate concentration at equilibrium (mg L^–1^); *K_F_* is the Freundlich adsorption capacity constant (mg^1‑(1/n)^) g^–1^ (L^1/n^); $\frac{1}{n}$ is the constant related to the surface heterogeneity.

The adsorption–desorption cycles were performed to evaluate biochar regeneration, adapting the methodology of Ghani et al.^65^ Experiments used 500 mg L^–1^ MB, 1.5 g L^–1^ A-BC2, at 25 °C for 1440 min under 105 rpm agitation. After each adsorption cycle, the biochar was separated by centrifugation (4000 rpm) and regenerated with 20 mL of 0.1 mol L^–1^ NaOH under agitation (180 rpm) for 30 min at 25 °C. The material was then rinsed with 10 mL Type II water under agitation for 10 min, centrifuged, and reused for the next cycle. This procedure was repeated for cycles 2 and 3.

The thermodynamic study of adsorption was performed through isotherm experiments at 25, 30, and 40 °C. From these data, the thermodynamic parameters, entropy (Δ*S*), enthalpy (Δ*H*), Gibbs free energy (Δ*G*°), and activation energy (Ea), were calculated using eqs 7–10. This analysis aimed to elucidate the predominant adsorption mechanisms.

7 $$\Delta G^{{}^{\circ}}=-R\times T\times \ln\left(K\right)$$

8 $$\Delta G^{{}^{\circ}}=\Delta H-\Delta S$$

9 $$\ln\left(K\right)=-\frac{\Delta H}{R}\times \frac{1}{T}+\frac{\Delta S}{R}$$

10 $$\ln\left(K\right)=-\frac{E_{a}}{2.303\times R}\times \frac{1}{T}+\ln\left(A\right)$$

Where Δ*H* (kJ mol^–1^) and Δ*S* (kJ K^–1^ mol^–1^) are the standard enthalpy and entropy changes of the adsorption process, respectively; Δ*G*° is the standard Gibbs free energy change (kJ mol^–1^); *T* is the absolute temperature (K); *K* is the equibibrium constant of the adsorption process (L mg^–1^); *R* is the universal gas constant (kJ K^–1^ mol^–1^); Ea is the activation energy (kJ mol^–1^); and *A* is the Arrhenius factor.

### 4.6 Statistical Analysis

To evaluate the results of the adsorption assays, a two-way analysis of variance (ANOVA) with replication was performed (*p* ≤ 0.05). The experimental data were fitted to the kinetic and isotherm models, and the most suitable model was selected based on the coefficient of determination (*R* ^2^), the adjusted *R* ^2^ (*R* ^2^ adj), the chi-square (χ^2^) and the ANOVA results.

## Supplementary Material



## Data Availability

All data are available in the text.

## Notes

Figure S1: XRD of the sewage sludge (BS) and activated biochars (A-BCs). A-BC1 (*T* = 400 °C); A-BC2 (*T* = 450 °C); A-BC3 (*T* = 500 °C); and A-BC4 (*T* = 550 °C). Figure S2: energy-dispersive X-ray spectra (EDS) of the biochars and the biological sludge. (a) A-BC1 (*T* = 400 °C); (b) A-BC2 (*T* = 450 °C); (c) A-BC3 (*T* = 500 °C); and (d) A-BC4 (*T* = 550 °C); (e) sludge; Figure S3: elemental mapping of A-BC1 obtained through energy-dispersive X-ray spectroscopy (EDS) analysis; Figure S4: elemental mapping of A-BC2 obtained through energy-dispersive X-ray spectroscopy (EDS) analysis; Figure S5: elemental mapping of A-BC3 obtained through energy-dispersive X-ray spectroscopy (EDS) analysis; Figure S6: elemental mapping of A-BC4 obtained through energy-dispersive X-ray spectroscopy (EDS) analysis; Figure S7: elemental mapping of sludge obtained through energy-dispersive X-ray spectroscopy (EDS) analysis; Figure S8: thermogravimetric analysis (TGA) and DTG of the biochars and the biological sludge. (a) A-BC1 (*T* = 400 °C); (b) A-BC2 (*T* = 450 °C); (c) A-BC3 (*T* = 500 °C); (d) A-BC4 (*T* = 550 °C); (e) sludge; Figure S9: determination of the point of zero charge (pHPZC) of the biochars. (a) A-BC1 (*T* = 400 °C); (b) A-BC2 (*T* = 450 °C); (c) A-BC3 (*T* = 500 °C); (d) A-BC4 (*T* = 550 °C). Figure S10: removal of methylene blue by the activated biochars (A-BCs): A-BC1 (*T* = 400 °C); A-BC2 (*T* = 450 °C); A-BC3 (*T* = 500 °C); A-BC4 (*T* = 550 °C), and the commercial activated carbon (AC). Experimental conditions: room temperature (∼25 °C), adsorption time 1444 min, agitation: ∼180 rpm, initial dye concentration: 100 mg L^‑1^, adsorbent dose: 1 g L^‑1^;, and natural solution pH; Figure S11: adsorption isotherms of methylene blue by A-BC2 at different temperatures. Experimental conditions: pH = 8, agitation: ∼180 rpm, and adsorbent dose: 1.50 g L^‑1^. Figure S12: schematic of the reactor used in the experiments. Table S1: mass percentage of chemical elements, disregarding carbon, from biochar and sludge based on EDS analysis. Determination of elemental composition in the biochars and sludge using Inductively Coupled Plasma Optical Emission Spectrometry (ICP-OES). Table S3: estimated production costs of the activated biochars A-BC1, A-BC2, A-BC3, and A-BC4 (PDF)
