# Supplementary material for: Sustainable Conversion of Pulp Industry Sludge into Activated Biochar for High-Performance Methylene Blue Removal
Source: ACS Omega. 2026 Jan 30;11(6):9497–509. doi: 10.1021/acsomega.5c09623 (PMC12917631; doi:10.1021/acsomega.5c09623)
Supplement: Supplementary file 1 [file ao5c09623_si_001.pdf]

Sustainable Conversion of Pulp Industry Sludge into Activated Biochar for High-Performance Methylene Blue Removal

Antonio Machado Netto [a], Marcela de Oliveira Brahim Cortez[a], José Pedro Rodrigues Ferreira[a], Renê Chagas da Silva[b], Leonarde Rodrigues [b], Luciano de Moura Guimarães [b], Renata Pereira Lopes Moreira[a]*

[a]Departament of Chemistry, Universidade Federal de Viçosa (UFV), Av. Peter Henry Rolfs, s/n, Campus Universitário, 36570-900 Viçosa-MG, Brasil.

[b]Departament of Physics, Universidade Federal de Viçosa (UFV), Av. Peter Henry Rolfs, s/n, Campus Universitário, 36570-900 Viçosa-MG, Brasil.

* renata.plopes@ufv.br (Corresponding author)

Table of contents

Figure S1. XRD of the sewage sludge (BS) and activated biochars (A-BCs). A-BC1 (T = 400°C); A-BC2 (T = 450°C); A-BC3 (T = 500 °C); A-BC4 (T = 550°C). 4

Figure S2. Energy-dispersive X-ray spectra (EDS) of the of the biochars and the biological sludge. (a) A-BC1 (T = 400 °C); (b) A-BC2 (T = 450 °C); (c) A-BC3 (T = 500 °C); (d) A-BC4 (T = 550 °C); (e) Sludge. 4

Figure S3. Elemental mapping of A-BC1 obtained through energy-dispersive X-ray spectroscopy (EDS) analysis..........................5

Figure S4. Elemental mapping of A-BC2 obtained through energy-dispersive X-ray spectroscopy (EDS) analysis..........................5

Figure S5. Elemental mapping of A-BC3 obtained through energy-dispersive X-ray spectroscopy (EDS) analysis..........................6

Figure S6. Elemental mapping of A-BC4 obtained through energy-dispersive X-ray spectroscopy (EDS) analysis..........................6

Figure S7. Elemental mapping of sludge obtained through energy-dispersive X-ray spectroscopy (EDS) analysis..........................7

Figure S8. Thermogravimetric analysis (TGA) and DTG of the biochars and the biological sludge. (a) A-BC1 (T = 400 °C); (b) A-BC2 (T = 450 °C); (c) A-BC3 (T = 500 °C); (d) A-BC4 (T = 550 °C); (e) Sludge. 7

Figure S9. Determination of the point of zero charge (pH_{PZC}) of the biochars. (a) A-BC1 (T = 400°C); (b) A-BC2 (T = 450°C); (c) A-BC3 (T = 500 °C); (d) A-BC4 (T = 550°C). 8

Figure S10. Removal of methylene blue by the activated biochars (A-BCs). A-BC1 (T = 400 °C); A-BC2 (T = 450 °C); A-BC3 (T = 500 °C); A-BC4 (T = 550 °C) and the commercial activated carbon (AC). Experimental conditions: room temperature (~25 °C), adsorption time 1444 min, agitation: ~180 rpm, initial dye concentration: 100 mg L^{-1}, adsorbent dose: 1 g L^{-1}, natural solution pH. 8

Figure S11. Adsorption isotherms of methylene blue by A-BC2 at different temperatures. Experimental conditions: pH = 8, agitation: ~180 rpm, and adsorbent dose: 1.50 g L^{-1}. 9

Figure S12. Schematic of the reactor used in the experiments. 9

Table S1. Mass percentage of chemical elements disregarding carbon from biochar and sludge from EDS analysis. 10

Table S2. Determination of elemental composition in the biochars and sludge using Inductively Coupled Plasma Optical Emission Spectrometry (ICP-OES).. 10

Table S3. Estimated production costs of the activated biochars A-BC1, A-BC2, A-BC3, and A-BC4. 11

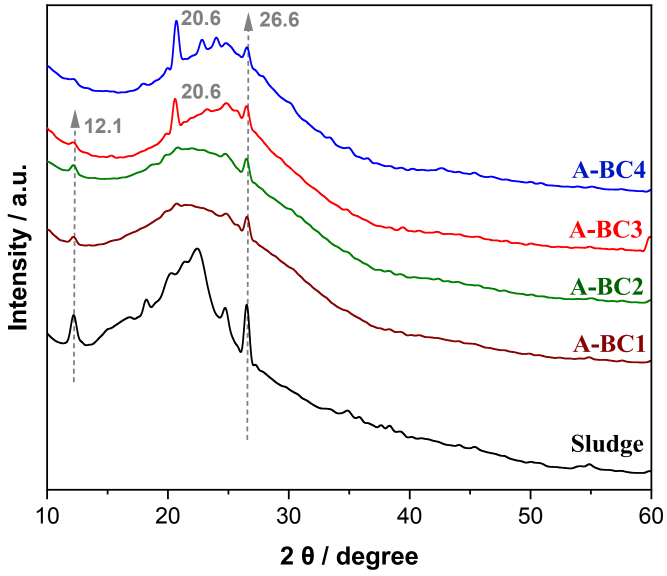

Figure S1. XRD of the sewage sludge (BS) and activated biochars (A-BCs). A-BC1 (T = 400°C); A-BC2 (T = 450°C); A-BC3 (T = 500 °C); A-BC4 (T = 550°C).

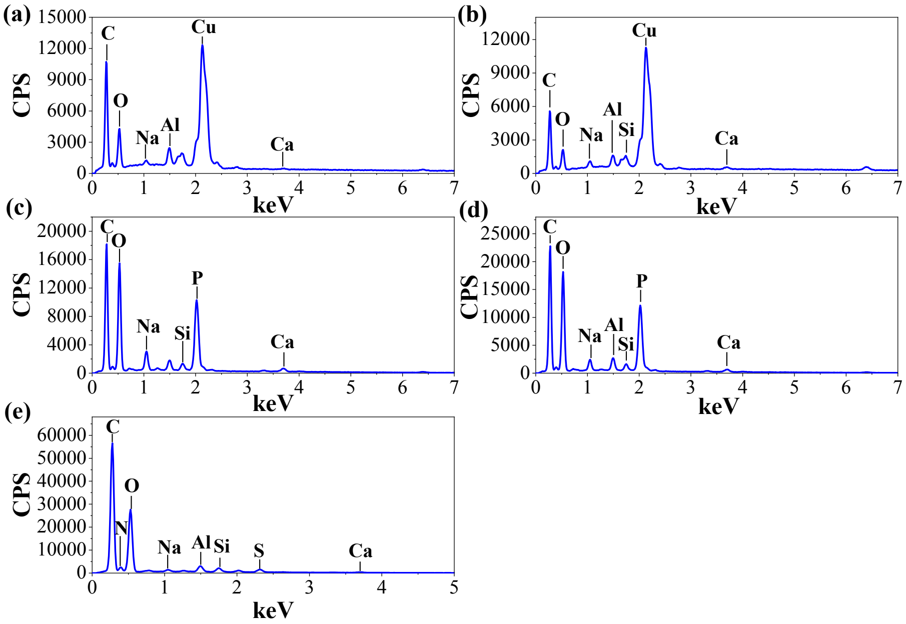

Figure S2. Energy-dispersive X-ray spectra (EDS) of the of the biochars and the biological sludge. (a) A-BC1 (T = 400 °C); (b) A-BC2 (T = 450 °C); (c) A-BC3 (T = 500 °C); (d) A-BC4 (T = 550 °C); (e) Sludge.

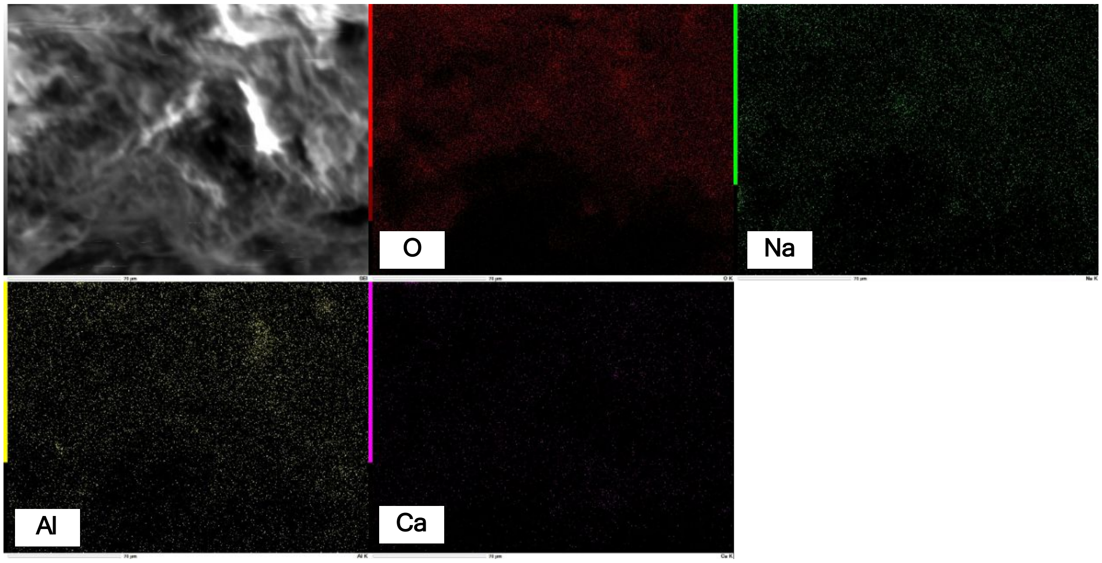

Figure S3. Elemental mapping of A-BC1 obtained through energy-dispersive X-ray spectroscopy (EDS) analysis.

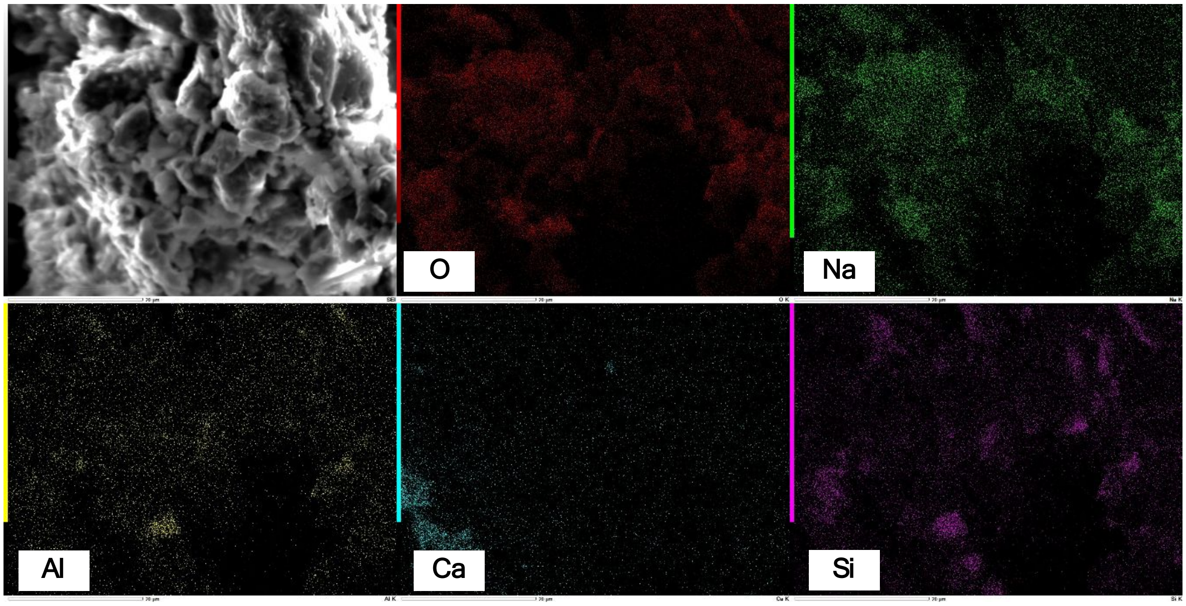

Figure S4. Elemental mapping of A-BC2 obtained through energy-dispersive X-ray spectroscopy (EDS) analysis.

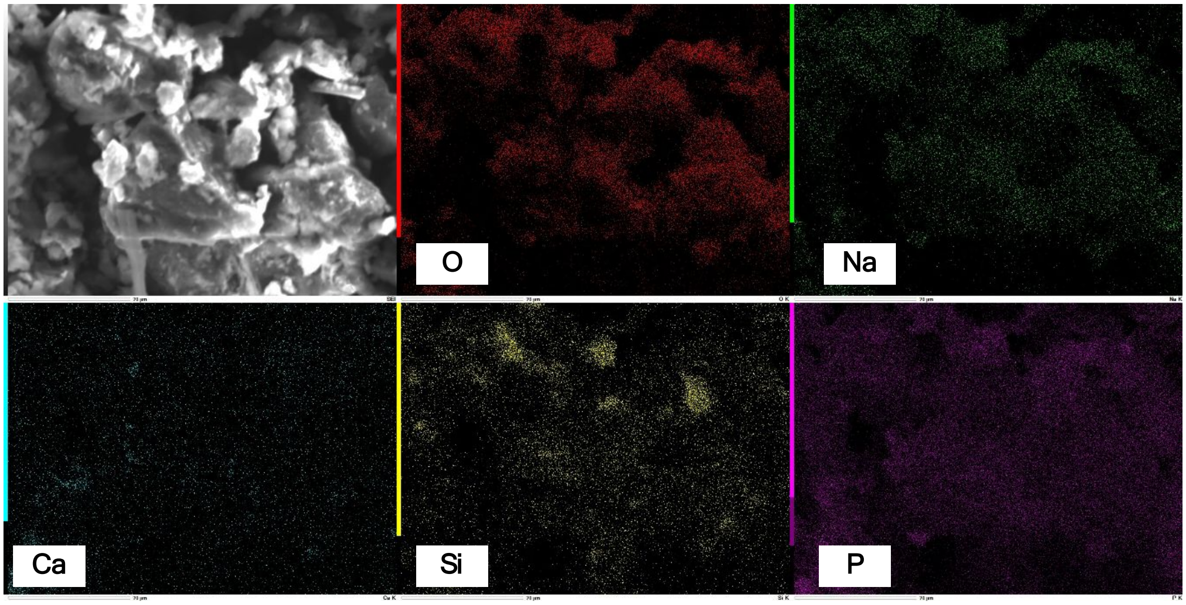

Figure S5. Elemental mapping of A-BC3 obtained through energy-dispersive X-ray spectroscopy (EDS) analysis.

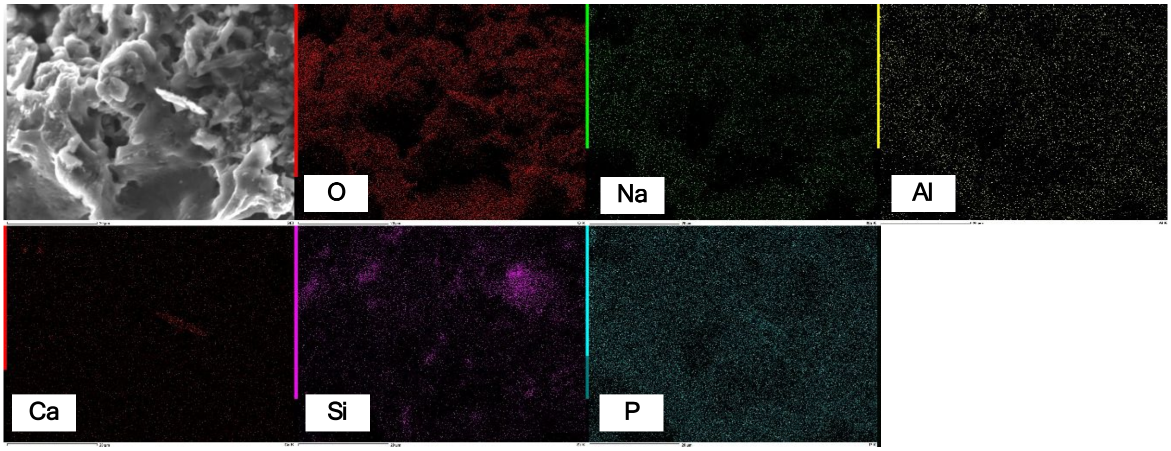

Figure S6. Elemental mapping of A-BC4 obtained through energy-dispersive X-ray spectroscopy (EDS) analysis.

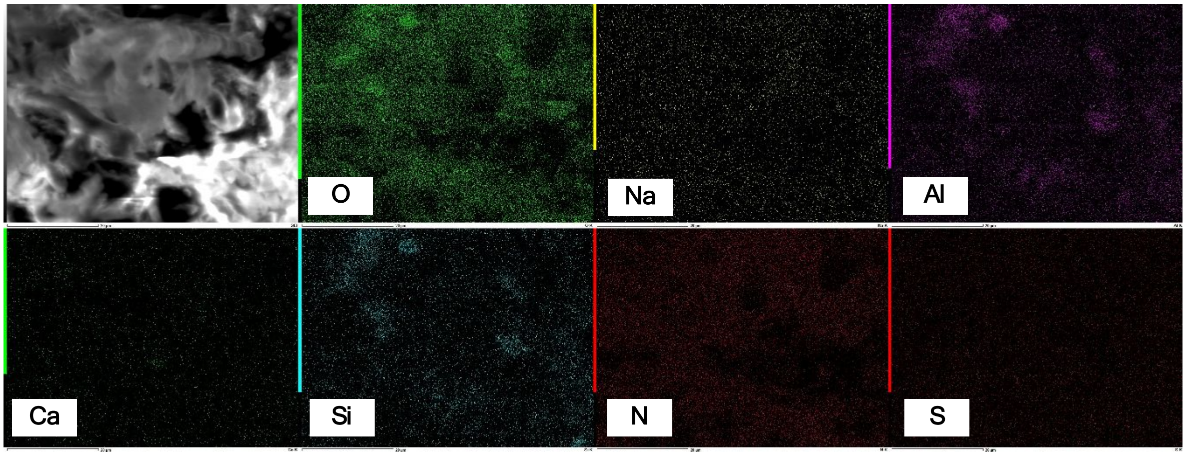

Figure S7. Elemental mapping of sludge obtained through energy-dispersive X-ray spectroscopy (EDS) analysis.

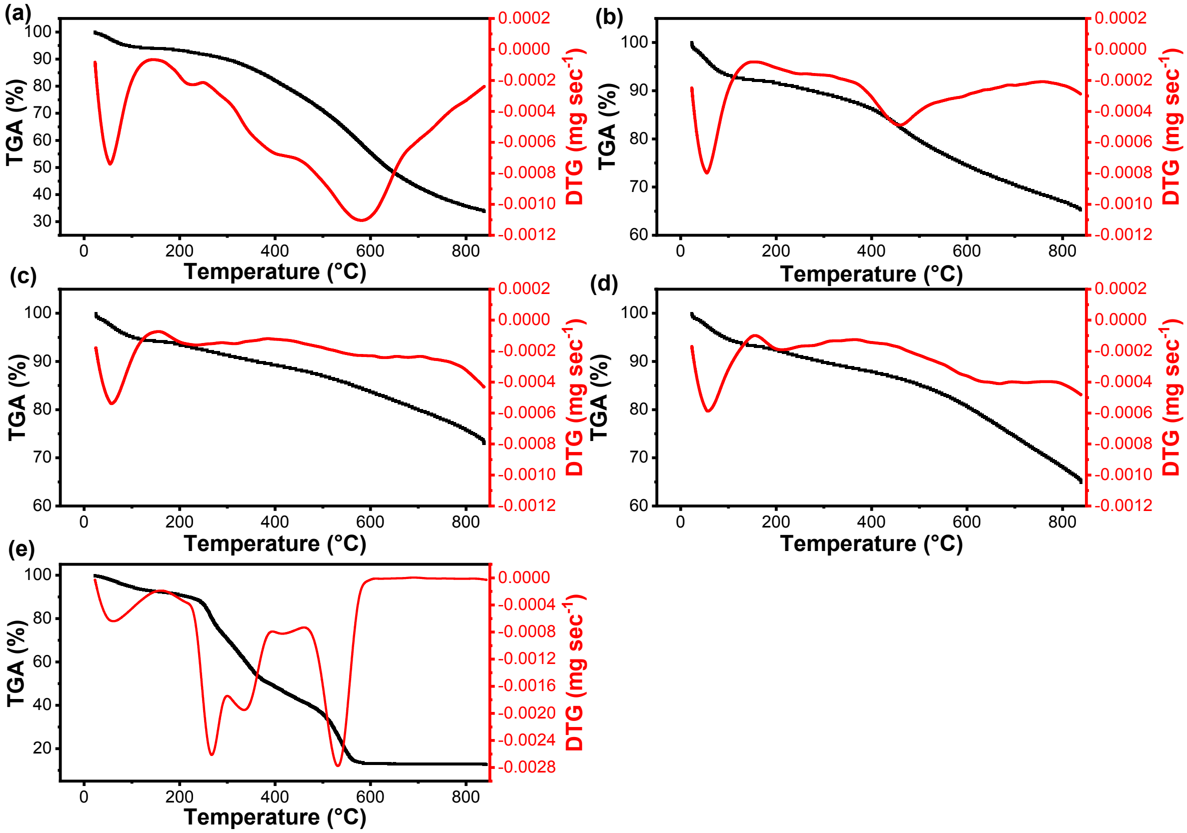

Figure S8. Thermogravimetric analysis (TGA) and DTG of the biochars and the biological sludge. (a) A-BC1 (T = 400 °C); (b) A-BC2 (T = 450 °C); (c) A-BC3 (T = 500 °C); (d) A-BC4 (T = 550 °C); (e) Sludge.

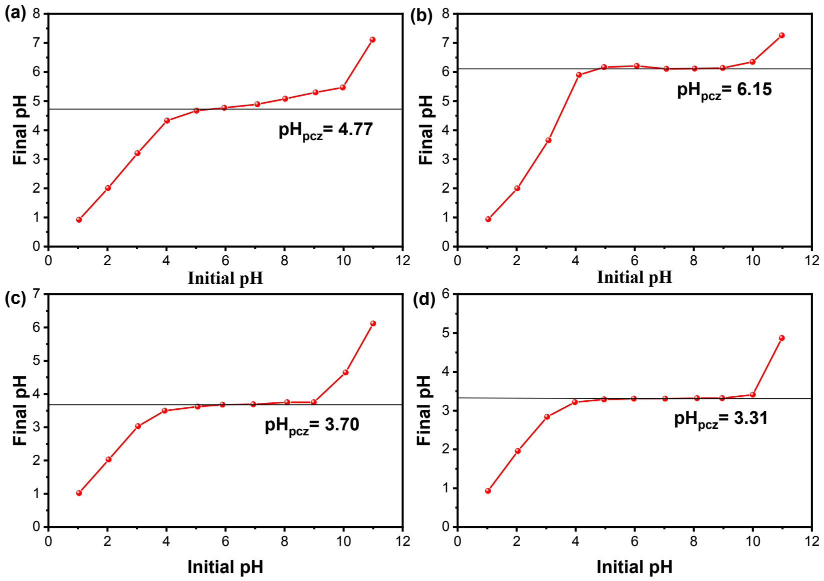

Figure S9. Determination of the point of zero charge (pH_{PZC}) of the biochars. (a) A-BC1 (T = 400°C); (b) A-BC2 (T = 450°C); (c) A-BC3 (T = 500 °C); (d) A-BC4 (T = 550°C).

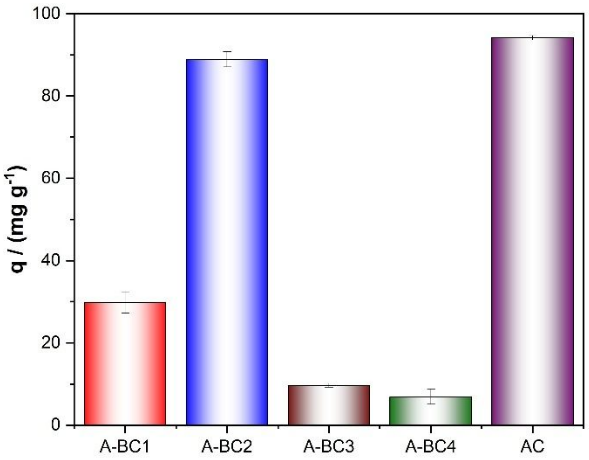

Figure S10. Removal of methylene blue by the activated biochars (A-BCs). A-BC1 (T = 400 °C); A-BC2 (T = 450 °C); A-BC3 (T = 500 °C); A-BC4 (T = 550 °C) and the commercial activated carbon (AC). Experimental conditions: room temperature (~25 °C), adsorption time 1444 min, agitation: ~180 rpm, initial dye concentration: 100 mg L^{-1}, adsorbent dose: 1 g L^{-1}, natural solution pH.

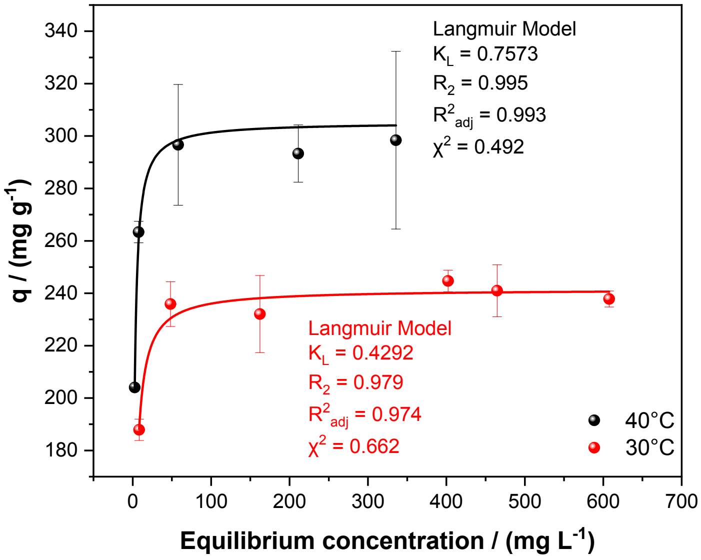

Figure S11. Adsorption isotherms of methylene blue by A-BC2 at different temperatures. Experimental conditions: pH = 8, agitation: ~180 rpm, and adsorbent dose: 1.50 g L^{-1}.

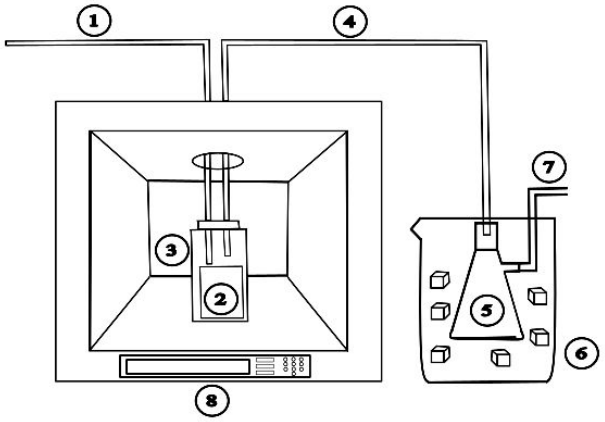

Figure S12. Schematic of the reactor used in the experiments. 1 - Nitrogen inlet tube; 2 - Stainless steel crucible; 3 - Pyrolysis cell; 4 - Gas outlet tube; 5 - Kitasato flask for collecting condensable gases; 6 - Ice bath; 7 - Outlet tube for non-condensable gases; 8 – Muffle furnace. Reproduced from Netto et al. [1], Tertiary Treatment of Pulp Industry Effluents Using Activated Biochar Derived from Biological Sludge Within a Circular Economy Framework, Processes, 13(6), Article 1647, 2025. CC BY 4.0 license.

Table S1. Mass percentage of chemical elements disregarding carbon from biochar and sludge from EDS analysis.

Chemical	Mass (%)				
	A-BC1	A-BC2	A-BC3	A-BC4	Sludge
O	78.3	67.97	64.60	63.81	75.11
Na	5.63	9.49	5.48	3.31	1.30
Al	14.92	12.42	ND*	3.27	3.34
Ca	1.15	3.55	2.33	1.89	0.92
Si	ND*	6.57	1.75	1.88	2.32
P	ND*	ND*	25.84	25.83	ND*
N	ND*	ND*	ND*	ND*	14.70
S	ND*	ND*	ND*	ND*	2.31
Total	100	100	100	100	100

ND* Not detectable

Table S2. Determination of elemental composition in the biochars and sludge using Inductively Coupled Plasma Optical Emission Spectrometry (ICP-OES).

Element	Concentration (mg g^{-1})				
	A-BC1	A-BC2	A-BC3	A-BC4	Slude
Al	< LoQ	0.017	0.089	0.030	0.045
Cd	< LoQ	< LoQ	< LoQ	< LoQ	< LoQ
Cr	< LoQ	< LoQ	< LoQ	< LoQ	< LoQ
Cu	< LoQ	< LoQ	< LoQ	< LoQ	< LoQ
Fe	< LoQ	0.841	0.636	0.035	< LoQ
Hg	< LoQ	< LoQ	< LoQ	< LoQ	< LoQ
K	< LoQ	< LoQ	< LoQ	< LoQ	< LoQ
Mg	< LoQ	0.186	0.195	0.163	0.137
Mn	< LoQ	< LoQ	< LoQ	< LoQ	< LoQ
Na	16.426	23.358	17.736	21.782	19.609
Ni	< LoQ	< LoQ	< LoQ	< LoQ	< LoQ
Pb	< LoQ	< LoQ	< LoQ	< LoQ	< LoQ
Si	0.044	0.081	0.101	0.066	0.097
Zn	< LoQ	< LoQ	< LoQ	< LoQ	< LoQ

LoQ = Limit of Quantification.

Table S3. Estimated production costs of the activated biochars A-BC1, A-BC2, A-BC3, and A-BC4.

Production Step	Description	Unit Cost (US$/unit)	Estimated production cost (US$)			
			A-BC1	A-BC2	A-BC3	A-BC4
Biomass acquisition	No cost, considering biochar production integrated into an existing industry	0	0.00	0.00	0.00	0.00
Activation agent (H_3PO_4)	(kg of H_3PO_4 / density) × cost per L of H_3PO_4 (1.17 US$ L^{-1})	1.17 US$ L^{-1} [2]	0.44	0.51	0.40	0.45
Activation	(Evaporated water (kg) / density (kg m^{-3})) × energy cost for evaporation process (US$ m^{-3})	4.98 US$ m^{-3} [2]	0.052	0.061	0.048	0.053
Nitrogen	Mass (kg) of N_2 used during 1 h of pyrolysis × nitrogen cost	0.114 US$ kg^{-1} [3]	0.00007	0.00007	0.00007	0.00007
Pyrolysis	Estimated energy required to heat activated biomass to pyrolysis temperature × electricity cost	0.1 US$ kwh^{-1} [4]	0.0771	0.0891	0.0711	0.0811
Basic washing	4 L NaOH (0.5 mol L^{-1}) × NaOH molar mass × NaOH price (US$ kg^{-1}) + water cost	0.144 US$ kg^{-1} [5]	0.0115	0.0115	0.0115	0.0115
Washing	Water consumption × process water cost	0.00053 US$ L^{-1} [6]	0.0000053	0.0000053	0.0000053	0.0000053
Drying	Water consumption during washing × energy cost for evaporation process	4.98 US$ m^{-3} [2]	0.07	0.07	0.07	0.07
	**Total cost (US$) per kg of biochar**		0.65	0.74	0.60	0.67
	**Total cost (US$) per g of biochar**		0.00065	0.00074	0.00060	0.00067

REFERENCES

[1] Netto AM, Nascimento MCGM, De Caux LS, Cortez MDOB, Ferreira JPR, Monteiro KA, et al. Tertiary Treatment of Pulp Industry Effluents Using Activated Biochar Derived from Biological Sludge Within a Circular Economy Framework. Processes 2025;13:1647. https://doi.org/10.3390/pr13061647.

[2] Gu H, Zhou G, Wen H, Wang N. Using of phosphoric acid to dissolve phosphate ore flotation tailings for stepwise separation of calcium and magnesium values. Chemical Engineering Science 2025;307:121356. https://doi.org/10.1016/j.ces.2025.121356.

[3] Pan X, Ma J, Hu X, Guo Q. Energy and economic analysis of a hydrogen and ammonia co-generation system based on double chemical looping. Chinese Journal of Chemical Engineering 2021;36:190–8. https://doi.org/10.1016/j.cjche.2020.10.007.

[4] Surmi A, Shariff AM, Lock SSM. Techno-economic assessment of cryogenic Rotating Packed Beds for nitrogen removal from natural gas. Results in Engineering 2025;26:104918. https://doi.org/10.1016/j.rineng.2025.104918.

[5] Davis R, Tao L, Tan E, Biddy M, Beckham G, Scarlata C, et al. Process Design and Economics for the Conversion of Lignocellulosic Biomass to Hydrocarbons: Dilute-Acid and Enzymatic Deconstruction of Biomass to Sugars and Biological Conversion of Sugars to Hydrocarbons. 2013. https://doi.org/10.2172/1107470.

[6] Peters MS, Timmerhaus KD, West RE, West RE. Plant design and economics for chemical engineers. 5. ed, international ed. 2004. Boston: McGraw-Hill; 2004.
